# Generation of human hepatocytes from extended pluripotent stem cells

**DOI:** 10.1038/s41422-020-0293-x

**Published:** 2020-03-09

**Authors:** Qiming Wang, Da Sun, Zhen Liang, Junyi Wang, Xinxing Zhong, Yulin Lyu, Junning Cao, Zhongqing Lin, Yuanyuan Du, Zhenchuan Miao, Shichun Lu, Cheng Li, Jun Xu, Yan Shi, Hongkui Deng

**Affiliations:** 1 School of Basic Medical Sciences, State Key Laboratory of Natural and Biomimetic Drugs, Peking University Health Science Center and the MOE Key Laboratory of Cell Proliferation and Differentiation, School of Life Sciences, Peking-Tsinghua Center for Life Sciences, Peking University, 100871 Beijing, China; 2 State Key Laboratory of Chemical Oncogenomics, School of Chemical Biology & Biotechnology, Peking University Shenzhen Graduate School, Shenzhen, 518055 Guangdong China; 3 School of Life Sciences, Center for Bioinformatics, Center for Statistical Science, Peking University, 100871 Beijing, China; 4 Department of Hepatobiliary Surgery, The First Medical Center, Chinese PLA General Hospital, 28 Fuxing Road, Haidian, 100853 Beijing, China; 5 Beijing Vitalstar Biotechnology Co., Ltd, 100000 Beijing, China; 6 Hangzhou Repugene Technology Co., Ltd, Hangzhou, 311121 Zhejiang China

**Keywords:** Stem-cell differentiation, Pluripotent stem cells

Dear Editor,

Human pluripotent stem cells have great potential for application in regenerative medicine, as they permit the generation of different lineages of functional cell types via directed differentiation.^1^ However, application of conventional human pluripotent stem cells is limited due to several factors, including heterogeneity in pluripotency, differentiation bias, relatively slow proliferation, and poor single-cell survival.^2^ To overcome these problems, extensive efforts have been made in recent years, including optimizing the culture conditions of conventional human pluripotent stem cells^3^ and deriving new human pluripotent cell types such as naive human pluripotent cell lines.^1^ Recently, our group established a new type of pluripotent stem cells designated as extended pluripotent stem (EPS) cells,^4^ which have both embryonic and extraembryonic developmental potential. Compared to known pluripotent stem cell types, these cells have superior differentiation potential, as demonstrated by single-cell chimeric assays in vivo. Furthermore, EPS cells can be long-term expanded through single-cell passaging with a high proliferation rate. These unique features of EPS cells make them valuable in biomedical applications, such as gene targeting and animal model generation.^5,6^ In particular, it is promising to apply human EPS cells in directed differentiation but has not yet been explored. In this study, we sought to use human EPS cells in directed differentiation by generating human hepatocytes from these cells. Our group has made great efforts in establishing directed differentiation protocols for generation of human functional hepatocytes from conventional human pluripotent stem cells.^7–9^ In this work, we demonstrated that human EPS cells could be efficiently differentiated into functional hepatocytes (EPS-Heps) through adding a pretreatment step before hepatic differentiation. Importantly, compared to hepatocytes derived from conventional human pluripotent stem cells, EPS-Heps transcriptionally more resemble primary human hepatocytes.

We first tried to differentiate EPS cells into hepatocytes with a protocol for differentiation of human embryonic stem cells (ESCs) towards hepatocytes.^7^ However, only a small number of ALB^+^/AFP^+^ hepatic progenitor-like cells were generated from the EPS cells (Supplementary Information, Fig. S1a). EPS cells have naive pluripotent features that are shared by epiblast cells in blastocysts from pre-implantation stage.^4^ Notably, increasing evidence has suggested that naive pluripotent stem cells in pre-implantation epiblasts do not respond directly to germ layer induction and that their differentiation competence to embryonic lineages is acquired during the early stages of post-implantation development.^10^ Based on these clues, to differentiate human EPS cells into hepatocytes, we first transiently induced them into an early post-implantation epiblast-like state^10^ and further generated hepatocytes using an optimized protocol that was based on our previous work^7^ (Fig. 1a). In the first step, we used TeSR™2 medium (primed stem cell culture medium) to pretreat EPS cells, which was referred to as stage 1 (S1). To investigate the state of EPS-derived S1 (EPS-S1) cells, we performed transcriptome analysis by RNA sequencing of EPS-S1 cells and iPSCs/ESCs, and human epiblasts at different stages of implantation were used as controls.^11^ Interestingly, we found that EPS-S1 cells were similar to epiblast cells during day 6 to day 8 of implantation, whereas iPSCs/ESCs showed transcriptional patterns similar to those of epiblast cells from day 10 to day 12 of implantation (Fig. 1b). We further examined the expression of several epiblast-specific genes that were expressed during implantation.^11^ We found that *SOX9*, *MSX2*, *PTN*, and *TKTL1*, which were highly expressed in epiblasts from day 6 to day 8, were also upregulated in EPS-S1 cells but not in iPSCs/ESCs. In addition, *GALNT3*, *PCSK9* and *CSRP1*, which were highly expressed in epiblasts from day 10 to day 12, were highly expressed in iPSCs/ESCs but not in EPS cells or EPS-S1 cells (Supplementary Information, Fig. S1b). These results suggested that at S1, EPS cells were converted to a state with molecular features shared by early post-implantation epiblast-like cells that are distinct from those of conventional human pluripotent stem cells.

**Fig. 1:**
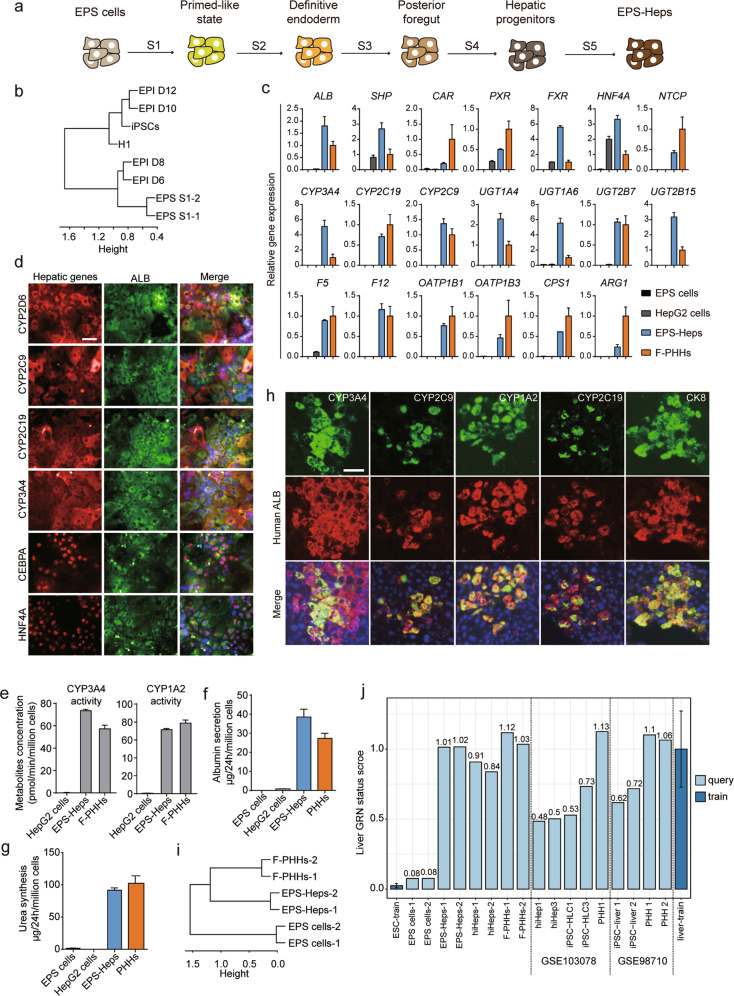
Generation of functional human hepatocytes from EPS cells. **a** Scheme of the five-stage protocol for the differentiation of EPS cells into human hepatocytes. **b** Hierarchical clustering of the gene expression profiles of epiblast cells (EPI) at different days, iPSCs, H1 ESCs and differentiated EPS cells at the end of stage 1 (EPS-S1 cells-1 and EPS-S1 cells-2). **c** RT-qPCR analysis of major hepatic genes and transcription factors in EPS cells (*n* = 3), HepG2 cells (*n* = 3), EPS-Heps matured for 5 weeks (*n* = 3) and F-PHHs (*n* = 5). Gene expression was normalized to F-PHHs and housekeeping gene. **d** Coimmunofluorescence staining of major hepatic functional markers and transcription factors with ALB in EPS-Heps. **e** UPLC/MS/MS analysis in HepG2 cells, EPS-Heps and F-PHHs for the drug metabolic activities of CYP3A4 and CYP1A2 by evaluating their metabolites, 6β-hydroxytestosterone and acetaminophen, respectively. *n* = 3. **f** ALB secretion in EPS cells, HepG2 cells, EPS-Heps matured for 4 weeks and PHHs analyzed by ELISA. PHHs were cultured for 5 days in vitro using sandwich method. *n* = 3. **g** Urea synthesis in EPS cells, HepG2 cells, EPS-Heps matured for 4 weeks and PHHs. PHHs were cultured for 5 days in vitro using sandwich method. *n* = 3. **h** Coimmunofluorescence staining of human ALB with human hepatic proteins, including CYP3A4, CYP2C9, CYP2C19, CYP1A2 and CK8, in URG mice transplanted with EPS-Heps matured for 8 weeks. **i** Hierarchical clustering of the gene expression profiles of EPS cells, F-PHHs and EPS-Heps. **j** The hepatic identities of EPS-Heps, F-PHHs, EPS cells, reprogrammed hepatocytes (hiHeps)^12^ and cells from other studies (GSE103078 and GSE98710) were analyzed by CellNet using RNA-seq data. Data are presented as means ± SEM. For all measurements, *n* represents the number of biological replicates. Scale bars, 50 μm. Original RNA-seq data of this paper are available in the NCBI Gene Expression Omnibus under accession number GSE137569.

After TeSR™2 pretreatment, we further treated the cells with signaling regulators of Activin/Nodal, WNT and PI3K/AKT and generated ~80% FOXA2^+^/SOX17^+^ definitive endoderm cells (Supplementary Information, Fig. S1c). Next, to initiate hepatic specification, we used our previously reported protocol for the hepatic specification of iPSCs/ESCs^7^ and found ~13% ALB^+^/AFP^+^ cells, indicative of hepatic progenitor cells (Supplementary Information, Fig. S1d). To increase the differentiation efficiency, we optimized our differentiation protocol of stage 4 to promote the generation of hepatic progenitors from posterior foregut cells. FACS analysis showed that ~70% of cells were ALB^+^/AFP^+^ cells (Supplementary Information, Fig. S1e). Characterization of these EPS-derived hepatic progenitor-like cells (EPS-HPLCs) showed the expression of key hepatic progenitor genes (Supplementary Information, Fig. S1f). Furthermore, immunofluorescence staining confirmed the expression of AFP and ALB in EPS-HPLCs (Supplementary Information, Fig. S1g). Notably, the global transcriptional profiles of EPS-HPLCs resembled those of human fetal liver cells (hFLCs) and were distinct from those of EPS cells (Supplementary Information, Fig. S1h). Collectively, these data suggested that hepatic progenitors could be efficiently induced from EPS cells in vitro.

Next, we aimed to generate functional EPS-derived hepatocytes (EPS-Heps) from the EPS-HPLCs using combination of a cAMP activator and a TGFβ inhibitor (hepatocyte maturation medium, HMM) that we recently developed to mature hepatic progenitors and maintain mature hepatocyte function.^12,13^ After maturation in HMM for 2 weeks, EPS-Heps showed a polygonal morphology that was similar to primary hepatocytes (Supplementary Information, Fig. S1i), which suggested that EPS-Heps with mature hepatic identity were generated. Therefore, we preformed molecular and functional test on EPS-Heps 4-5 weeks post maturation in HMM. RT-qPCR data showed that multiple hepatic transcription factors and functional genes, such as *HNF4A*, *PXR*, *CYP3A4*, *UGT2B7* and *NTCP*, were expressed in EPS-Heps, and the expression of all these genes were comparable to that of freshly isolated primary human hepatocytes (F-PHHs) (Fig. 1c; Supplementary Information, Fig. S2a). Immunofluorescence staining also confirmed the expression of CYP2D6, CYP2C9, CYP2C19, CYP3A4, CEPBA and HNF4A (Fig. 1d). Furthermore, the CYP3A4 and CYP1A2 activities of EPS-Heps were also comparable to those of F-PHHs evaluated by ultra-performance liquid chromatography tandem mass spectrometry (UPLC/MS/MS) (Fig. 1e). Importantly, the expression of CYP3A4 in EPS-Heps was inducible when EPS-Heps were exposed to the PXR agonist (rifampin) (Supplementary Information, Fig. S2b). In addition, the secretion of human albumin (ALB) in EPS-Heps gradually increased from 4 to 35 µg/day/million cells within the first 4 weeks of maturation (Supplementary Information, Fig. S2c). The ALB secretion, urea synthesis levels and the bile acid secretion of EPS-Heps were comparable to those of primary human hepatocytes (Fig. 1f, g; Supplementary Information, Fig. S2d). We also observed that EPS-Heps could uptake indocyanine green (ICG) and exclude it after withdrawal (Supplementary Information, Fig. S2e). Furthermore, the glycogen synthesis in EPS-Heps was detected by Periodic Acid-Schiff (PAS) staining (Supplementary Information, Fig. S2f). These data suggested that EPS-Heps acquired hepatic function.

We next investigated the repopulation capacity of EPS-Heps in vivo and transplanted EPS-Heps into Tet-uPA (urokinase-type plasminogen activator)/Rag2^−/−^/γc^−/−^ (URG) mice, a liver injury mouse model.^14^ By immunofluorescence staining, we detected the engraftment of human ALB^+^ cells in the mouse liver (Fig. 1h). The engrafted EPS-Heps were also functional, which was indicated by the co-expression of human CYP3A4, CYP2C9, CYP1A2 and CYP2C19 with human ALB (Fig. 1h). We also detected the secretion of human ALB in the serum of URG mouse repopulated with EPS-Heps (Supplementary Information, Fig. S2g). Furthermore, we analyzed the repopulation rate of engrafted URG mouse livers, and it was ~2% (Supplementary Information, Fig. S2h). These data suggested that EPS-Heps could repopulate the damaged mouse liver with functionally mature features.

Finally, to evaluate EPS-Heps at the global transcriptional level, we performed RNA-seq on EPS-Heps. As controls, F-PHHs and EPS cells were also analyzed. Importantly, hierarchical clustering revealed that EPS-Heps clustered closely with F-PHHs but apart from EPS cells (Fig. 1i). To further investigate the hepatic identity of EPS-Heps, we analyzed our RNA-seq data using CellNet, an algorithm that evaluates the fidelity of cells generated in vitro by comparing to their counterparts from native tissue.^15^ The CellNet gene regulatory network (GRN) status score and heatmap showed that EPS-Heps recapitulated hepatic identity, which was indistinguishable from F-PHHs (Fig. 1j; Supplementary Information, Fig. S2i). Similar results could be obtained from two different EPS cell lines in additional three different batches of experiments (Supplementary Information, Fig. S2j). We also noticed partial intestinal identity, which was due to the expression of intestine-related transcription factors such as *KLF5* in EPS-Heps. These off-target identities could be altered with further optimization of differentiation protocol. Additionally, using CellNet, we analyzed reprogrammed human induced hepatocytes (hiHeps)^12^ and public RNA-seq data of conventional iPSC-derived hepatocytes (GSE103078 and GSE98710). The results showed that the EPS-Heps more closely resembled F-PHHs than hepatocytes generated from conventional human pluripotent stem cells or from lineage reprogramming (Fig. 1j; Supplementary Information, Fig. S2i).

In summary, we established a differentiation protocol to efficiently generate functional hepatocytes from EPS cells, demonstrating the feasibility of applying EPS cells in directed differentiation. Importantly, the GRN of EPS-Heps was highly similar to that of primary human hepatocytes compared with that of hepatocytes generated from conventional human pluripotent stem cells. Notably, the successful induction of functional EPS-Heps was dependent on the pretreatment of EPS cells at S1, which induced these cells into a state having shared molecular features with human epiblast cells at early post-implantation stages. Furthermore, EPS-S1 cells were transcriptionally distinct from conventional human iPSCs/ESCs that more resemble the epiblast cells from late post-implantation stages (Fig. 1b; Supplementary Information, Fig. S1b). Therefore, it is possible that the early post-implantation features of EPS-S1 cells could contribute to better establishment of a hepatic transcriptional network of EPS-Heps compared with hepatocytes derived from conventional human pluripotent stem cells. Our study also suggests that current differentiation protocols for conventional human pluripotent stem cells could be adapted to the directed differentiation of EPS cells. Our results highlight the great applicative potential of EPS cells to generate functional cells, which could be a better cell source for the generation of desired cell types in vitro in the future.

## Supplementary information

Supplementary information, Figures and Materials
