# Supplementary material for: Generation of human hepatocytes from extended pluripotent stem cells
Source: Cell Res. 2020 Mar 9;30(9):810–3. doi: 10.1038/s41422-020-0293-x (PMC7608418; doi:10.1038/s41422-020-0293-x)
Supplement: Supplementary file 1 — Supplementary information, Figures and Materials [file 41422_2020_293_MOESM1_ESM.pdf]

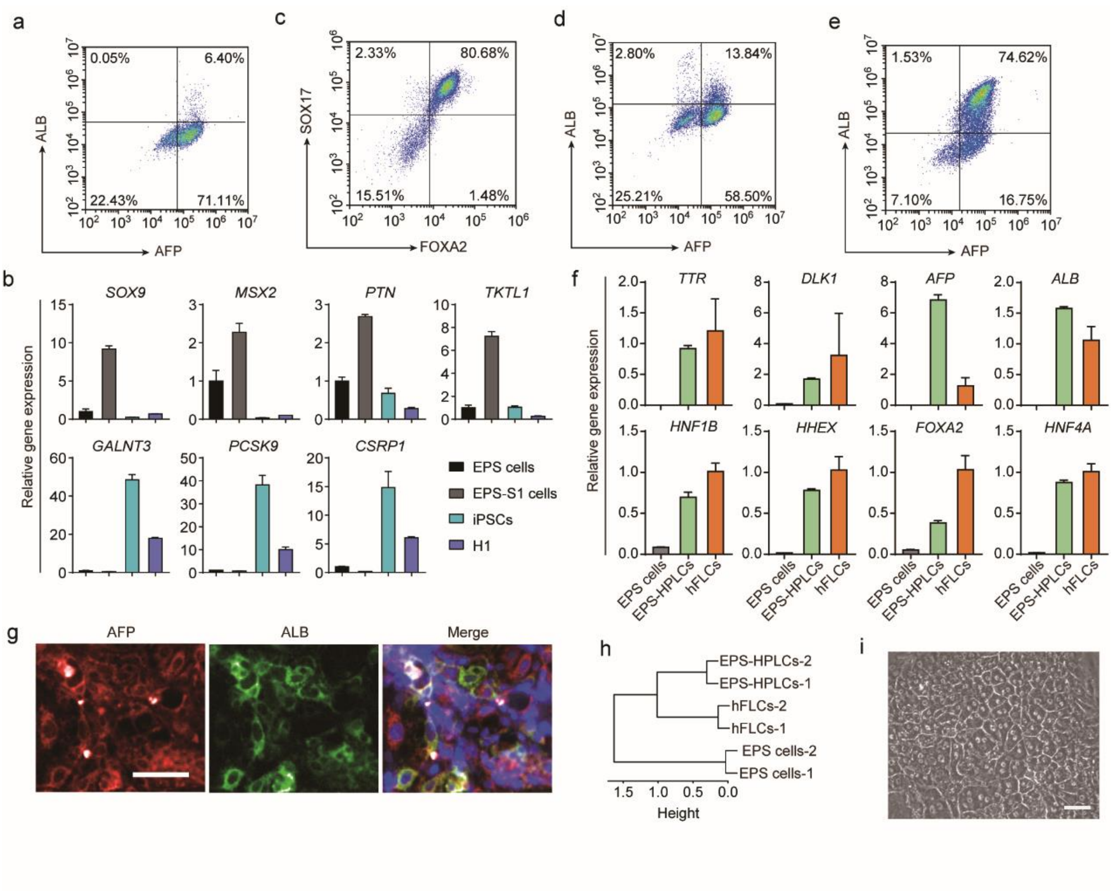

Supplementary information, Figure S1 Generation of hepatocytes from EPS cells *in vitro*.

(a) Flow cytometry analysis of AFP^+/ALB^+ EPS-derived hepatic progenitor cells without the treatment of S1 stage. (b) RT-qPCR analysis of epiblast genes in EPS cells, EPS-S1 cells, iPSCs and H1 ESCs. n = 3. (c) Flow cytometry analysis of $FOXA2^+/SOX17^+$ definitive endoderm cells showed the differentiation efficiency at the end of stage 2 (S2). (d) Flow cytometry analysis of AFP^+/ALB^+ hepatic progenitor cells differentiated from TeSR™2-treated EPS cells with previously reported protocol for differentiation of human embryonic stem cells. (e) Flow cytometry analysis of AFP^+/ALB^+ hepatic progenitor cells showed the differentiation efficiency at the end of stage 4 (S4). (f) RT-qPCR analysis of human hepatic progenitor markers in EPS cells, EPS-HPLCs and hFLCs. Gene expression was normalized to hFLCs and housekeeping

gene. n = 3. (g) Coimmunofluorescence staining of ALB and AFP in EPS-HPLCs. (h) Hierarchical clustering of the gene expression profiles of EPS cells, hFLCs and EPS-HPLCs. (i) Morphology of EPS-Heps shown by bright field images. The data are presented as the mean ± SEM. For all measurements, ‘n’ represents the number of biological replicates. The scale bars represent 50 μm.

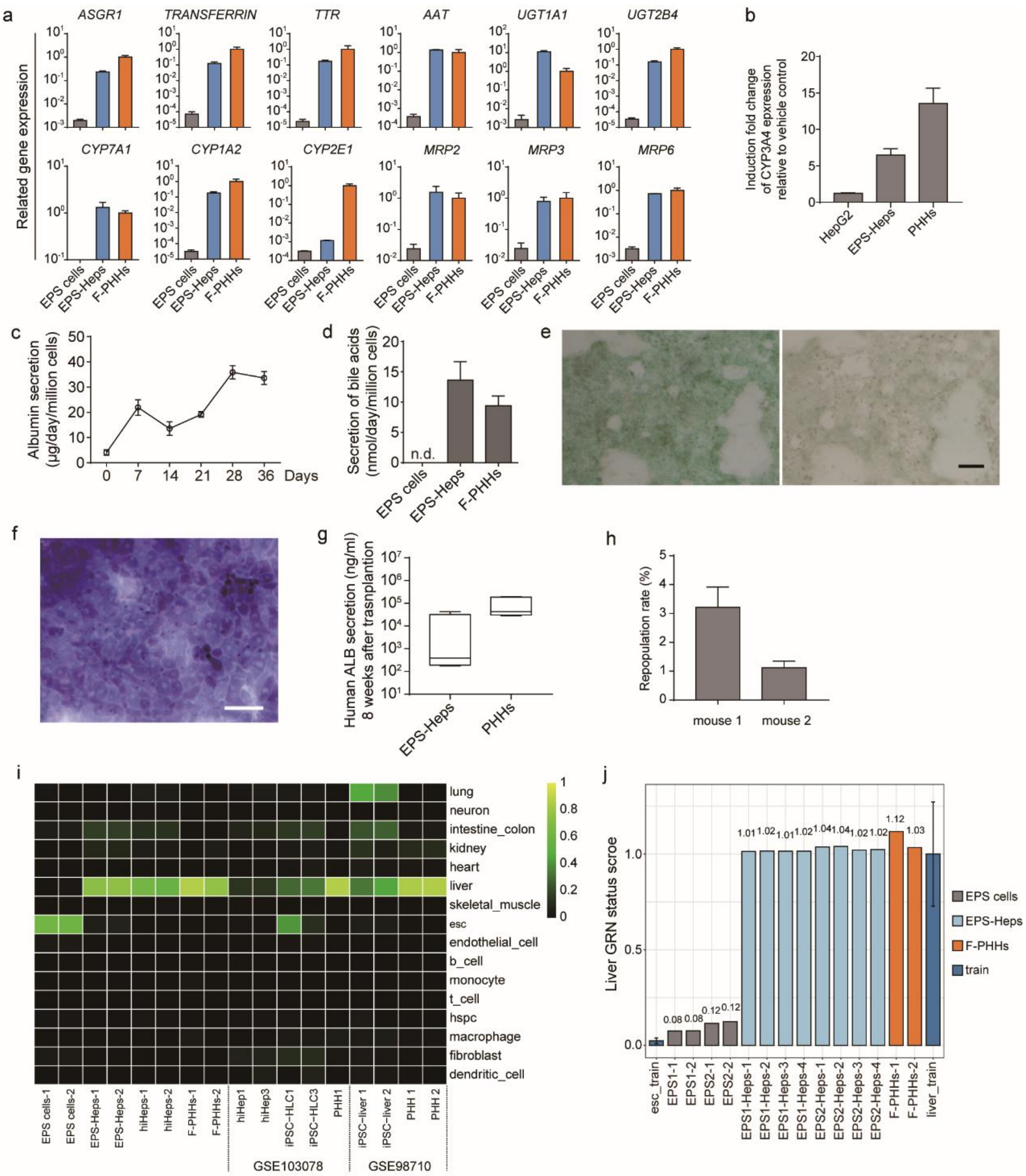

Supplementary information, Figure S2 Characterization of EPS-derived Hepatocytes.

(a) RT-qPCR analysis of human hepatic functional genes in EPS cells (n=3), EPS-Heps (n=3) and F-PHHs (n = 3). Gene expression was normalized to F-PHHs and housekeeping gene. (b) RT-qPCR analysis of induction fold change of *CYP3A4* expression after a three-day treatment of PXR ligand (50 μM rifampin) in HepG2 cells (n = 3), EPS-Heps (n = 3) and PHHs (n = 2).

(c) Dynamic analysis of ALB secretion in EPS-Heps since day 0 post maturation in hepatic maturation medium. n = 3. (d) Bile acids secretion in EPS cells, EPS-Heps and PHHs. n = 3. (e) Uptake (left) and release (right) of ICG in EPS-Heps. The scale bars represent 100 μm. (f) PAS staining of EPS-Heps. The scale bars represent 50 μm. (g) Human ALB secretion in URG mice serum after the transplantation of EPS-Heps (n = 4) and PHHs (n = 5). (h) The repopulation rate of EPS-Hep-engrafted URG mice livers were determined by human ALB immunostaining 8 weeks post transplantation. n = 6 for each mouse. Data were presented as mean ± SEM. (i) The cell identity heatmap of EPS-Heps, F-PHHs, EPS cells, reprogrammed hiHeps[12] and cells from other studies (GSE103078 and GSE98710) analyzed by CellNet using RNA sequencing data. (j) The hepatic identities of EPS cells, F-PHHs and EPS-Heps generated from 2 different EPS cell lines in 4 different batches of experiments, analyzed by CellNet using RNA sequencing data. The sample EPS1-1, EPS1-2, EPS1-Heps-1 and EPS1-Heps-2 is the same as EPS cells-1, EPS cells-2, EPS-Heps-1 and EPS-Heps-2 showed in Fig. 1j, respectively. Data are presented as the mean ± SEM. For all measurements, 'n' represents the number of biological replicates.

Materials and Methods

Cells and cell culture

Primary human hepatocytes were isolated as previously described. Briefly, human liver tissue was first perfused with PBE buffer (9 g/L NaCl, 0.42 g/L KCl, 2.1 g/L $NaHCO_3$, 0.9 g/L glucose, 4.78 g/L HEPES and 0.37 g/L EDTA in sterilized water) for 0.5 to 2 hours, and the liver tissue was further perfused with PBCD buffer (9 g/L NaCl, 0.42 g/L KCl, 2.1 g/L NaHCO3, 0.9 g/L glucose, 4.78 g/L HEPES, 0.25 g/L collagenase and 0.25 g/L dispase in sterilized water) until the liver tissue was incompact. The loosened liver tissue was separated with tweezers, and the human hepatocytes were collected for further experiments.

Human fetal liver tissue was obtained from aborted tissue with informed patient consent. The fetal liver tissue was shredded and incubated in 1 mg/ml collagenase IV for 20 minutes at 37°C. The tissue fragments and cells were plated on Matrigel-coated dishes in hepatic progenitor expansion medium, and the medium was changed every day. Hepatic progenitor expansion medium included 50% DMEM/F12 (Thermo Fisher Scientific), 50% William's E Medium (Thermo Fisher Scientific) supplemented with 1% penicillin-streptomycin (P/S, Thermo Fisher Scientific), 2% B27 (without VA, Thermo Fisher Scientific), 5 mM nicotinamide (Sigma-Aldrich), 200 μM 2-phospho-L-ascorbic acid (pVc, Sigma-Aldrich), 3 μM CHIR99021(Tocris), 5 μM SB431542 (Selleck), 0.5 μM sphingosine-1-phosphate (S1P, Sigma-Aldrich), 5 μM lysophosphatidic acid (LPA, Aladdin), 50 ng/ml EGF (Peprotech) and 20 μM forskolin (Tocris). After the hepatic progenitors migrated out from the fetal liver tissue and the cells were confluent, the cells and tissue fragments were digested with Accutase (Millipore). The tissue fragments were discarded with a sifter. The hepatic progenitors were collected for further experiments.

HepG2 cells were a gift from Kuanhui Xiang (Peking University Health Science Center) and were cultured in DMEM containing 10% FBS (Thermo Fisher Scientific), 1% GlutaMAX (Thermo Fisher Scientific), 1% P/S and 1% MEM NEAA (Thermo Fisher Scientific) and passaged with 0.25% trypsin (Thermo Fisher Scientific) at a ratio of 1:3.

The human ES cell line H1 was obtained from the WiCell Research Institute (Madison, WI, USA). Human iPS cell line was established using an episomal iPSC reprogramming kit (Invitrogen). iPS cells and H1 embryonic stem cells were cultured in TeSR™2 (STEMCELL Technologies) on Matrigel-coated plates and passaged with 50 mM EDTA (Thermo Fisher Scientific) at a ratio of 1:6 to 1:8.

The EPS cells used in this study were EPS1 cells and EPS2 cells, as previously described[1].

Generation of hepatocytes from EPS cells

EPS cells were cultured on mitomycin C (Sigma-Aldrich) inactivated mouse embryonic fibroblast (MEF) feeder cells (3×10^4 cells per cm^2) with EPS culture medium (48.25% DMEM/F12 (Thermo Fisher Scientific), 48.25% Neurobasal medium (Thermo Fisher Scientific), 0.5% N2 supplement (Thermo Fisher Scientific), 1% B27 supplement (Thermo Fisher Scientific), 1% GlutaMAX, 1% MEM NEAA, 1% P/S, 10 ng/ml recombinant human LIF (PeproTech), 1 μM CHIR99021, 2 μM (S)-(+)-dimethindene maleate (Tocris) and 2 μM minocycline hydrochloride (Santa Cruz Biotechnology). The EPS cells were passaged using 0.05% trypsin-EDTA (Thermo Fisher Scientific) at a proportion from 1:3 to 1:10.

To induce EPS cells into a primed liked state in stage 1, EPS cells were digested into single cells with 0.05% Trypsin-EDTA and seeded at 5×10^4 cells/cm^2 in TeSR™2 medium for 48

hours on Matrigel-coated plates. To generate definitive endoderm in stage 2, differentiated cells were treated with MCDB medium (Thermo Fisher Scientific) containing 1% B27 (without vitamin A), 100 ng/ml Activin A (Stemimmune LLC), 0.25 mM pVc, 25 ng/ml Wnt3a (R&D), and 0.05 μM PI103 (Selleck) for 1 day and were then treated with MCDB medium containing 100 ng/ml Activin A and 0.25 mM pVc for 3 days. Next, to generate posterior foregut in stage 3, the differentiated cells were cultured in modified MCDB medium containing 1% B27 (without vitamin A), 50 ng/ml KGF (Stemimmune LLC), 0.25 mM pVc and 10 μM SB431542 (Selleck) for 3 days. Next, in stage 4, to generate hepatic progenitors from the anterior foregut cells, the differentiated cells were first cultured in DMEM medium (Thermo Fisher Scientific) containing 1% B27 (without vitamin A), 20 ng/ml KGF, 10 ng/ml bFGF (Peprotech), 20 ng/ml BMP2 (Stemimmune LLC) and 50 ng/ml BMP4 (Stemimmune LLC) for 4 days. Next, the cells were replated at a ratio of 1:3 and cultured in modified William's E Medium (Beijing Vitalstar Biotechnology) for 8 days. After that, the cells were further cultured in hepatic expansion medium (HEM) containing 49% William's E Medium, 49% DMEM/F12, 2% B27, 0.25 mM pVc, 5 μM SB431542, 3 μM CHIR99021, 0.5 μM S1P, 5 μM LPA, 40 ng/ml EGF and 10 mM Nicotinamide for at least 10 days. In this stage, the cells were passaged when they were confluent. In stage 5, hepatocyte maturation medium (HMM), which contained William's E Medium, 2% B27, 1% GlutaMAX, 10 μM SB431542 and 50 μM forskolin, was used to generate mature hepatocytes from hepatic progenitors. These EPS-Heps could be matured and maintained in HMM. All media were changed every day. Accutase was used for passaging in all stages. All the molecular and functional test of EPS-Heps were preformed 4 to 5 weeks post maturation.

RT-qPCR analysis

Total RNA was isolated with RNeasy Mini Kit (QIAGEN) following the manufacturer's instructions. RNA (500 ng) was reverse-transcribed to cDNA with TransScript First-Strand cDNA Synthesis SuperMix (TransGen Biotech). KAPA SYBR® FAST Universal qPCR Mix (KAPA Biosystems) was used for RT-qPCR analysis, which was performed on a BIO-RAD CFX384™ Real-Time System. All relative expression levels were normalized to the housekeeping gene RRN18S. The RT-qPCR primer sequences are provided in the following table.

Gene	Forward Primer (5' to 3')	Reverse Primer (5' to 3')
RRN18S	GTAACCCGTTGAACCCCATT	CCATCCAATCGGTAGTAGCG
TTR	TAGGAGTAGGGGCTCAGCAG	TAGGAGTAGGGGCTCAGCAG
HHEX	ACGGTGAACGACTACACGC	CGTTGGAGAATCTCACCTGG
FOXA2	CGACTGGAGCAGCTACTATGC	TACGTGTTCATGCCGTTCAT
HNF1B	GCACCTCTCCCAGCATCTCA	GTCGGAGGATCTCTCGTTGC
DLK1	GGGCACAGGAGCATTCATAG	GACGGGGAGCTCTGTGATAG
SHP	AGGGACCATCCTCTTCAACC	ACTTCACACAGCACCCAGTG
ALB	GCACAGAATCCTTGGTGAACAG	ATGGAAGGTGAATGTTTCAGCA
AFP	CCCGAACTTTCCAAGCCATA	TACATGGGCCACATCCAGG
UGT1A6	AATTTCCTAAAGGCCGGTCA	TTGATCCCAAAGAGAAAACCA
UGT2B15	GTTTTCTCTGGGGTCGATGA	ATTTGGCTTCTTGCCATCAA
F5	ACAGCGTCGTCCATCTTCTC	GATAAGCCCTTGAGCATCCA

F12	TGGTACTGGAAGGGGAAGTG	CTTTCGATTCCACCTTGGG
OATP1B1	TTCAATCATGGACCAAAATCAA	TGAGTGACAGAGCTGCCAAG
OATP1B3	GAAAACAAGACGCTGCAATG	TCCTTTCTATTTGAGTGATGGAAA
CPS1	AATGAGGTGGGCTTAAAGCAAG	AGTTCCACTCCACAGTTCAGA
ARG1	GTGGAAACTTGCATGGACAAC	AATCCTGGCACATCGGGAATC
CYP2C19	GAAGAGGAGCATTGAGGACCG	GCCCAGGATGAAAGTGGGAT
CYP2C9	GCCACATGCCCTACACAGATG	TAATGTCACAGGTCACTGCATGG
CYP3A4	AGCCTGGTGCTCCTCTATCT	CCCTTATGGTAGGACAAAAT
NTCP	AGGGGGACATGAACCTCAG	AGGTCCCCATCATAGATCCC
UGT1A4	AACGGGAAGCCACTATCTCA	TCAGCAATTGCCATAGCTTTC
UGT2B7	AACGTAATTGCATCAGCCCT	GGTCATTCTGGGGTATCCAC
CAR	TTGCAGAAGTGCTTAGATGCTG	GCCGACAGTATCATGTCTTTCCT
FXR	CCTGTGAGGGGTGTAAAGGTT	CACTCTTGACACTTTCTTCGCAT
PXR	AAGCCCAGTGTCAACGCAG	AGATTTGGGGACCTCCGACTT
HNF4A	CGTGGTGGACAAAGACAAGA	CATAGCTTGACCTTCGAGTGC
SOX9	AGCGAACGCACATCAAGAC	CTGTAGGCGATCTGTTGGGG
MSX2	ATGGCTTCTCCGTCCAAAGG	CGGCTTCTTGTCGGACATGA
PTN	GGAGCTGAGTGCAAGCAAAC	CTCGCTTCAGACTTCCAGTTC
TKTL1	ACAAGCAGTCAGATCCAGAGA	TAGCTGGCCCTGTCGAAGTA
GALNT3	CAGCAGAATTGAAGCCTGTCC	CTTCCCCACGTTCCTTTTCCT
PCSK9	AGACCCACCTCTCGCAGTC	GGAGTCCTCCTCGATGTAGTC

CSRP1	TGCCGAAGAGGTTCAGTGC	AGCAGGACTTGCAGTAAATCTC
CYP1A2	CTTCGCTACCTGCCTAACCC	GACTGTGTCAAATCCTGCTCC
CYP2E1	GACCACCAGCACAACTCTGA	CCCAATCACCCTGTCAATTT
CYP7A1	TGTCCTGGAAGATTGTTCGCT	GGACATTTAGCTTGGCCCTCT
MRP2	GGGATCTCTTCCACACTGGAT	CATACAGGCCCTGAAGAGGA
MRP3	AGGCCAGCAGGGAGTTCT	AGCTCGGCTCCAAGTTCTG
MRP6	AAGAACTTGTTTCCCGGCTT	CTCGGTCTCTGGAGCCTTC
UGT1A1	CCATCATGCCCAATATGGTT	CCACAATTCCATGTTCTCCA
UGT2B4	TCTTTCGATCCCAACAGCC	CATCTCTTAACCAGCTGCTTGATA
ASGR1	GAGAGAGACGTTCAGCAACTTC	GGGACTCTAGCGACTTCATCTT
TRANSFERRIN	GTGTGCAGTGTCGGAGCAT	CATCGGATGGAATGACGCTTT
TTR	TAGGAGTAGGGGCTCAGCAG	TAGGAGTAGGGGCTCAGCAG
AAT	ATGCTGCCCAGAAGACAGATA	CTGAAGGCGAACTCAGCCA

Albumin ELISA, urea assay, bile acids secretion, PAS staining and ICG assay.

Human albumin in the cell supernatant or in the mouse serum were measured using Human Albumin ELISA Quantitation Kit (Bethyl Laboratory) according to the manufacturer's instructions. Urea and bile acids in the cell supernatant was measured using QuantiChrom Urea Assay Kit (BioAssay Systems) and Bile Acid Assay Kit (Sigma-Aldrich) according to the manufacturer's instructions, respectively. The cell supernatant samples were collected and stored at -20°C. To evaluate the glycogen storage on EPS-Heps, the Periodic Acid-Schiff (PAS)

staining was performed using PAS staining kit (Sigma-Aldrich). EPS-Heps were fixed with 4% paraformaldehyde (DingGuo) and stained according to manufacturer's instructions. To test if EPS-Heps could uptake indocyanine green (ICG), EPS-Heps were culturd in 1 mg/ml ICG diluted in HMM for 1 hour. The images were captured immediately or 6 hour after ICG was released.

Immunofluorescence Staining

For immunofluorescence staining, cells were fixed with 4% paraformaldehyde (DingGuo) for 15 minutes and blocked with PBST (0.25% Triton X-100 and 5% normal donkey serum in PBS). Then, the cells were incubated with primary antibodies at 4°C overnight followed by the appropriate secondary antibodies for 1 hour at room temperature. DAPI (Roche) was used to indicate nuclei. The information of antibodies are listed in the following table.

Gene	Antibody	Catalog Number
FOXA2	Hu FOXA2 PE N17-280	561589 (BD Pharmingen)
SOX17	Hu SOX17 Alexa 647 P7-969	562594 (BD Pharmingen)
ALB	Human Albumin Antibody	A80-129A (Bethyl Laboratories, Inc.)
AFP	Alpha-1-Fetoprotein Antibody	ZM-0009 (ZSGB-BIO)
CYP3A4	CYTOCHROME P450 3A4	AHP622Z (BIO-RAD)
CYP2C9	CYTOCHROME P450 2C9	AHP617Z (BIO-RAD)
CYP2C19	CYTOCHROME P450 2C19	AHP618Z (AbD Serotec)

CYP2D6	Rabbit anti Human CYP2D6	HPA045223 (Sigma-Aldrich)
CK8	Mouse anti Human CK8	ZM-0310 (ZSGB-BIO)
HNF4A	HNF4A Antibody	sc-8987 (Santa Cruz)
CEBPA	CEBPA Rabbit Antibody	2295s (Cell Signaling Technology)
HNF6A	HNF6A Antibody	sc-13050 (Santa Cruz)
DLK1	DLK (N-18) Antibody	sc-8623 (Santa Cruz)
Second antibody	Donkey Anti-Mouse IgG 488	A-21202 (Invitrogen)
Second antibody	Donkey Anti-Goat IgG 555	A-21432 (Invitrogen)
Second antibody	Donkey Anti-Rabbit IgG 488	A-21206 (Invitrogen)

Flow Cytometry

For flow cytometry analysis, cells were released into single-cell suspensions with Accutase and fixed with Fixation/Permeabilization solution (BD) for 20 min at 4°C. Then, the cells were incubated with primary antibodies diluted in 1X BD Perm/Wash buffer at 4°C for 2 hours followed by the appropriate secondary antibodies for 1 hour at 4°C. Finally, the cells were re-suspended in BD Perm/Wash buffer and analyzed on a CytoFLEX (Beckman Coulter) flow cytometry system. The data were analyzed with CytExpert software. The antibodies used in flow cytometry analysis were the same as those used for immunofluorescence staining.

RNA sequencing and bioinformatic analysis

We assessed the quality of the raw RNA sequencing data with FastQC software. The raw fastq files were trimmed with Trimmomatic software using the following parameters: ILLUMINACLIP:/path/to/adapters/TruSeq3-PE-2.fa:2:30:7:1:true LEADING:3 TRAILING:3 SLIDINGWINDOW:4:15 HEADCROP:10 MINLEN:36. The trimmed clean data were then aligned to the human reference genome hg19 with STAR software with the default parameters. Next, the gene count matrix of all samples was generated with the featureCounts function of the R package Rsubread. Finally, normalization and variance-stabilizing transformation were performed on the gene count matrix with the R package DESeq2. The differentially expressed genes were also determined with DESeq2.

To compare our transcriptome data with the annotated epiblast single-cell RNA sequencing data (GSE109555), we reanalyzed the public data with the R package Seurat and simulated the bulk RNA sequencing data of epiblast cells by averaging the gene expression levels of all epiblast cells in the same day.

We performed hierarchical clustering with the R function hclust. The distance between two samples was defined as one minus the Pearson correlation between the z-scores of the gene vectors. The sample distance definition above was also used to draw heatmaps with the R package pheatmap.

We performed CellNet analysis on our data and some public RNA sequencing data (GSE103078 and GSE98710). The RNA sequencing data were analyzed with the R package CellNet. We followed the official CellNet pipeline to analyze all the sequencing data and visualize the results.

Animals and Transplantation

Tet-uPA/Rag2$^{-/-}$/$\gamma c^{-/-}$ (URG) mice on a BALB/c background were purchased from Beijing Vitalstar Biotechnology. For transplantation, cells were dissociated to single cells with Accutase and suspended in HCM^{TM} medium (Lonza) at a final concentration of 10^7 cells/ml. URG mouse was injected with 200 μl suspension into the spleen. Eight weeks post injection, mouse was sacrificed for immunofluorescence staining. The liver of the mouse was fixed with 4% paraformaldehyde and dehydrated with a 30% sucrose solution. Then, the liver tissue was embedded in OCT compound (Sakura) and frozen in liquid nitrogen. Cryosections were generated using a cryostat (Leica) for immunofluorescence staining. The repopulation rate of human ALB positive cells was evaluated with Vectra Polris (PerkinElmer) using 6 random cryosections.

The experiment on mouse model was approved by the Institutional Animal Care and Use Committee of Peking University, and were performed according to NIH guidelines.

Measurements of CYP3A4 and CYP1A2 Activities

EPS-Heps, HepG2 cells and freshly isolated primary human hepatocytes were dissociated and suspended to measure their CYP3A4 and CYP1A2 activities. One 500 μL reaction contained 2.5×10^5 cells and 200 μM testosterone or 200 μM phenacetin as substrate of CYP3A4 or CYP1A2, respectively. Reaction without cells as well as reaction without testosterone or phenacetin were also performed to exclude reaction background. After incubation for 15 minutes at 37 °C, reactions were stopped by 1.5 mL methanol containing

isotope-labeled reference metabolite, 6β-hydroxytestosterone-[D7] for CYP3A4 or Acetomidophenol-[13C2, 15N] for CYP1A2, for further mass spectrometry (ultra-performance liquid chromatography-tandem mass spectrometry, UPLC/MS/MS) analysis. The drug-metabolic product of CYP3A4 and CYP1A2 reaction for UPLC/MS/MS analysis were 6β-Hydroxytestosterone and Acetaminophen, respectively.
